# The Association Between Statin Therapy and the Subsequent Clinical Course of Patients With Melioidosis

**DOI:** 10.1155/jotm/8838580

**Published:** 2025-05-25

**Authors:** Laura Prideaux, Hayley Stratton, Meg Sandeman, Simon Smith, Josh Hanson

**Affiliations:** ^1^Department of Medicine, Cairns Hospital, Cairns 4870, Queensland, Australia; ^2^The Kirby Institute, University of New South Wales, Kensington 2033, New South Wales, Australia

## Abstract

**Background:** Even in well-resourced settings, the case-fatality rate of melioidosis approaches 10%. This has prompted an interest in identifying adjunctive therapies that might improve survival. A prospective, multicentre study in Thailand suggested that statin therapy may reduce the incidence of pneumonia in patients with melioidosis; however, the impact of statins on the clinical course of patients with the infection is incompletely defined.

**Materials and Methods:** We examined all cases of culture-confirmed melioidosis in Far North Queensland, tropical Australia, since October 2016 to determine if statin therapy influenced the clinical phenotype of melioidosis and the patients' clinical course.

**Results:** Of 321 individuals with culture-confirmed melioidosis, 100 (31%) were prescribed a statin at the time of their diagnosis. There was no difference in the clinical phenotype of patients who were— and were not—taking statin therapy. Pulmonary involvement, specifically, was no less common in patients taking a statin (79/100 [79%] versus 175/221 [79%], *p* = 0.97). A smaller proportion of patients taking statin therapy died before hospital discharge, but this difference did not reach statistical significance (5/100 [5%] versus 26/221 [12%], *p* = 0.07). This finding was at least partially explained by the fact that fewer patients with an active malignancy were taking a statin (7/37 [19%] versus 93/284 [33%] patients without a malignancy, *p* = 0.09) and that, in multivariable analysis, patients with malignancy were more likely to die before hospital discharge (odds ratio [95% confidence interval]: 4.73 [1.62–13.87], *p* = 0.005). Among 290 individuals surviving to hospital discharge, there was no difference in 12-month mortality between those that were—and were not—prescribed a statin at presentation (11/95 [12%] versus 23/195 [12%], *p* = 0.96).

**Conclusion:** Statin therapy does not appear to have any significant influence on the clinical phenotype of patients with melioidosis. There is also no appreciable impact of statin therapy on patients with melioidosis' short-term or 12-month survival.

## 1. Introduction

Melioidosis, a life-threatening infection caused by the environmental Gram-negative bacterium *Burkholderia pseudomallei*, is emerging as a major global health threat [1, 2]. Modelling suggested that already in 2015, there may have been 165,000 melioidosis cases globally, with 89,000 deaths [3]. A growing worldwide burden of the comorbidities that predispose individuals to developing melioidosis and an expanding geographic distribution of *B. pseudomallei* have the potential to increase the global incidence of the disease further [1, 4].

Melioidosis has a high case-fatality rate which can exceed 40% in resource-limited settings [1, 5, 6]. However, even in well-resourced health systems, about 10% of patients will die from their infection [7, 8]. Over 20% of individuals with melioidosis in tropical Australia present with septic shock and approximately 25% require admission to the intensive care unit (ICU) [9, 10]. However, after the delivery of targeted antibiotic therapy, aggressive source control and optimal critical care support, there are no therapies that have shown to improve survival of these patients. While granulocyte colony stimulating factor (G-CSF) appeared to reduce mortality in observational studies, a randomised controlled trial showed no benefit [9, 11]. Australian guidelines recommend the addition of trimethoprim/sulfamethoxazole to patients with neurological infection, osteomyelitis, septic arthritis, genitourinary infection or skin and soft tissue infection, although randomised controlled trials have not demonstrated increased survival with this strategy [12, 13]. The significant rate of adverse reactions to trimethoprim/sulfamethoxazole also limits its utility in many patients [14, 15].

Statins (or 3-hydroxy-3-methyglutaryl coenzyme A reductase inhibitors) have an established role in the management of hyperlipidaemia and cardiovascular disease [16]. However, there has also been growing interest in the use of statins for other conditions, particularly in the management of infectious diseases [17]. Statins are inexpensive, widely available and are generally well tolerated, and it has been suggested that statins' anti-inflammatory, antioxidative, antithrombotic and immunomodulatory properties may have a salutary effect on the clinical course of patients with infection [17]. Moreover, as the most common predisposing risk factor for melioidosis globally is diabetes, many patients with melioidosis are already taking statin therapy [1].

Although randomised controlled trials of statins in patients with bacterial sepsis have shown no survival benefit [18], a recent multicentre prospective cohort study in Thailand reported that patients taking statins at presentation were less likely to develop pneumonia [19]. This is a potentially significant finding as most patients with melioidosis will have pneumonia and it is the clinical manifestation that is most commonly associated with septic shock [7, 20]. It is important to validate this finding as, if statins are truly protective against pneumonia, they may have utility as an adjunctive therapy in patients with— and at risk of—melioidosis.

In Far North Queensland (FNQ) in tropical Australia, diabetes is the most common predisposing risk factor for melioidosis, and many patients are already receiving statin therapy for primary or secondary prophylaxis of cardiovascular events. We performed this study to determine if this statin therapy had any impact on the clinical phenotype or clinical course of patients presenting with melioidosis in the region. We also examined the surviving patients' postdischarge course to determine if the prescription of a statin had any impact on long-term outcomes.

## 2. Materials and Methods

This study was conducted in accordance with the Declaration of Helsinki and received ethical approval from the FNQ Human Research Ethics Committee (HREC/15/QCH/46–977). As the data were deidentified, the ethics committee waived the requirement for individual patient consent.

The study was conducted at Cairns Hospital, a 531-bed tertiary referral hospital located in FNQ, a region of approximately 380,000 km^2^ in the northeastern most tip of tropical Australia. The hospital serves a population of approximately 290,000 people, of whom approximately 17% identify as Aboriginal or Torres Strait Islander Australians (hereafter respectfully referred to, collectively, as First Nations Australians). The Cairns Hospital laboratory is the reference laboratory for all microbiology services in the public health system in the FNQ region.

All cases with culture-confirmed *B. pseudomallei* infection diagnosed in this laboratory between 1 October 2016 and 30 April 2024 were eligible for inclusion in the study [21]. This period was chosen as it corresponded with the prospective collection of clinical data of the patients diagnosed with melioidosis. The patients' electronic medical records were reviewed for the patients' demographics, presentation, clinical phenotype, and clinical course, as described previously [22]. When individuals register with the public health system, they are routinely asked whether they identify as a First Nations Australian; this was documented. Socioeconomic disadvantage was defined using the Socioeconomic Indexes for Area (SEIFA) Index of Relative Socioeconomic Advantage and Disadvantage (IRSAD) score, the Australia Bureau of Statistics' measure of socioeconomic disadvantage [23]. Individuals were said to have lung involvement if they had any evidence of acute infection on chest imaging, or if they had *B. pseudomallei* cultured from sputum.

Pre-existing risk factors for melioidosis—including diabetes mellitus, hazardous alcohol use, chronic kidney disease, chronic lung disease, malignancy and immunosuppression—were actively sought and recorded, as described previously [22]. In FNQ, patients who present with melioidosis are routinely evaluated for all these comorbidities [24]. If patients had none of these six predisposing factors, they were said to have no risk factors for melioidosis. Patients with symptoms ≤ 2 months prior to admission were said to have an acute presentation; patients with symptoms for > 2 months were said to have a chronic presentation. Disseminated infection was defined as ≥ 3 noncontiguous infected sites, with bacteraemia not considered a distinct site of infection. Patients were said to have recurrent melioidosis if they had recrudescence (confirmed recurrence during targeted antimicrobial therapy), relapse (confirmed recurrence after completing eradication therapy) or reinfection (confirmed infection with a new, genomically unrelated strain or a confirmed infection greater than 2 years after the initial infection).

Data were deidentified, entered into a password-protected electronic database (Microsoft Excel) and analysed with statistical software (Stata Version 18.0). Groups were analysed using the chi-squared tests and logistic regression, where appropriate. Multivariable analysis was performed using backwards stepwise logistic regression and included all variables that had a *p* value < 0.10 in univariable analysis. Survival data were analysed using a Cox proportional hazards model and presented using Kaplan–Meier curves. If individuals were missing data, they were not included in analyses which evaluated those variables. Socioeconomic disadvantage was presented using the decile of the SEIFA IRSAD score of the patients' residential address, with the first decile indicating greatest disadvantage [23].

## 3. Results

There were 321 cases of culture-confirmed melioidosis during the study period. The patients' median (interquartile range [IQR]) age was 57 (46–69) years; 212 (66%) were male and 130 (41%) identified as a First Nations Australian. Overall, 290/321 (90%) survived to hospital discharge. The factors associated with survival to hospital discharge are presented in Table 1. In multivariable analysis, patients were more likely to die in hospital if they had septic shock (odds ratio [OR]; 95% confidence interval [CI]: 13.93 [5.82–33.30], *p* < 0.001) or had malignancy (OR [95% CI]: 4.73 [1.62–13.87], *p*=0.005).

### 3.1. Characteristics and Clinical Course of the Patients

Statins were prescribed to 100/321 (31%) at the time of their presentation. Patients prescribed statin therapy were older and were more likely to have diabetes mellitus, macrovascular disease or chronic kidney disease. A lower proportion of patients with an active malignancy were prescribed statin therapy, but this difference did not reach statistical significance (Table 2).

There was no difference in the clinical phenotype of the patients that were and were not prescribed statin therapy. Specifically, there was no difference in the proportion of patients with lung involvement. A lower proportion of patients prescribed statin therapy died before hospital discharge, although this difference did not reach statistical significance in either univariable or multivariable analysis (Tables 1 and 2).

**Figure 1:**
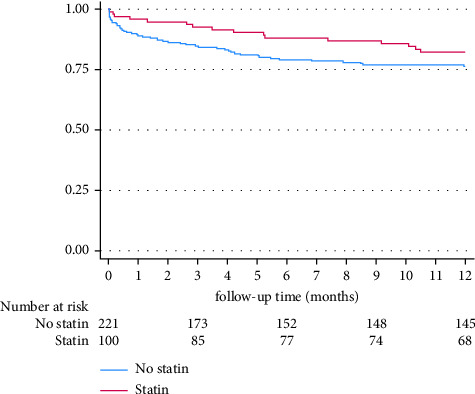
Kaplan–Meier curve comparing the all-cause mortality of individuals who survived to hospital discharge with melioidosis in the 12-month postdiagnosis, who were—and were not—prescribed statin therapy at presentation.

Of 290/321 (90%) who survived to discharge, 95 (33%) were prescribed a statin. There was no difference in all-cause mortality at 12 months between the patients that were—and were not—prescribed statin therapy (OR [95% CI]: 0.98 [0.46–2.10], *p*=0.96) (Figure 1).

## 4. Discussion

Almost a third of the patients in this cohort were prescribed a statin when they were diagnosed with melioidosis, but, in contrast to the findings of a prior Thai study, the prescription of a statin appeared to have no impact on the clinical phenotype of the infection. Although a lower proportion of individuals prescribed a statin at presentation died before hospital discharge, this is likely to be at least partly explained by the fact that fewer patients with an active malignancy were receiving statin therapy [25, 26]. There was also no reduction in mortality at 12 months in the patients who were prescribed a statin and who survived their hospitalisation with melioidosis.

A growing understanding of the pathophysiology and clinical presentation of melioidosis—and significant improvements in the management of patients with sepsis—has led to a dramatic decline in the case-fatality rate of patients with melioidosis in well-resourced settings [1, 9, 27]. There is interest in the development of adjunctive therapies that may reduce this case-fatality rate further [28]. A multicentre cohort study in Thailand demonstrated that patients taking statins were less likely to develop melioidosis-associated pneumonia [19]. This was a potentially important finding as pneumonia is present in over 70% of cases of melioidosis in some series and it is the clinical manifestation of melioidosis that is most commonly associated with septic shock [7, 20]. However, in our study, there was no difference in the frequency of lung involvement in patients who were—and were not—prescribed statin therapy. There was also no impact on meaningful clinical endpoints including the incidence of septic shock or ICU admission.

These findings are reminiscent of early cohort studies that suggested that statin therapy may reduce the rates of pneumonia [29, 30], the rate of sepsis [31, 32] and have a positive effect on short- and long-term mortality of patients with these conditions [33–36]. Only for multiple randomised controlled trials to show no significant benefit from statin use in patients with sepsis or pneumonia, no inflammatory rebound in patients in whom this therapy was ceased and the potential for hepatic and renal toxicity in some patients who were taking statins [18, 37–43]. The disparity in findings between these cohort studies and the randomised controlled trials has, as in our study, been felt most likely to be explained by the confounding effects of healthy user bias [40, 42].

It was concerning to note that most individuals with established macrovascular disease and almost a third of people living diabetes were not prescribed statin therapy, despite the proven benefits of statin therapy in these patient groups [16, 44]. Melioidosis is a disease of socioeconomic disadvantage, and in the FNQ region, First Nations Australians—particularly those living in remote communities—are disproportionately affected [22, 45]. The fact that many patients in the cohort in whom statin therapy would be recommended highlights that even in Australia's well-resourced universal health system, it is challenging to deliver optimal primary care to culturally diverse populations in regional and remote settings [46, 47]. As more Australian patients survive their hospitalisation with melioidosis, there is a growing recognition that the hospitalisation provides an opportunity for clinicians to optimise the care of the comorbidities that may have predisposed them to develop melioidosis and which may contribute to an increased risk of premature—and potentially preventable—death [48, 49]. This study provides data to suggest that many patients are not receiving this comprehensive care.

Our study has many limitations. Although we were able to record whether patients were prescribed a statin, in this retrospective cohort study, it was not possible to determine whether patients were actually taking the therapy; this may underestimate any potential benefit [50]. Patients took a variety of statins—at a variety of doses—and our analysis did not control for this, although a variety of statins have been trialled in randomised controlled trials in other critically ill populations and no benefit has been identified [51–53]. Some patients—particularly those who were critically unwell—may have had been unable to receive their statin therapy, or alternatively had it interrupted during their hospitalisation due to concerns about toxicity, which again may have affected our findings. We were not able to access the medical records of patients in primary care after their discharge, a period when patients not taking statin therapy may have had it commenced and those taking therapy may have had it ceased; our 12-month mortality data, therefore, need to be interpreted cautiously. Statin therapy is, of course, only one component of comprehensive care of patients at higher risk of macrovascular disease; our incomplete data in this small heterogeneous cohort do not suggest that statin therapy does not have a beneficial effect in patients with established indications.

However, acknowledging these caveats, we did not identify any signal to suggest that statin therapy has any impact on the clinical phenotype or clinical course of melioidosis. This finding is consistent with randomised controlled trials of pneumonia and sepsis due to other pathogens.

## 5. Conclusions

Even in well-resourced settings, approximately one in 10 individuals who develop melioidosis will succumb to their infection. At present there is no vaccine for this life-threatening but neglected tropical infectious disease and there is no adjunctive therapy that has proven to be effective [1]. Although a large, prospective Thai study suggested that the use of statin therapy reduced the risk of pneumonia in patients with melioidosis, our study found no evidence of any protective effect.

The quest, therefore, continues for diagnostic and therapeutic options that reduce the case-fatality rate of melioidosis. Until then, gains are only likely to be made by stringent application of evidence-based guidelines for the management of bacterial sepsis and by developing public health strategies that reduce the burden of the comorbidities that predispose individuals to developing this emerging, life-threatening infection [1, 2, 27, 45].

## Figures and Tables

**Table 1:** Characteristics of the patients who were associated with death before hospital discharge.

Variable	Died in hospital *n* = 31	Survived to hospital discharge *n* = 290	OR (95% CI)	*p* ^a^
Age (years)	60 (47–78)	57 (46–69)	1.01 (0.99–1.03)	0.43
Male sex	20 (65%)	192 (66%)	0.93 (0.43–2.01)	0.85
First Nations Australians	9 (29%)	121 (42%)	0.57 (0.25–1.28)	0.18
Remote residence	4 (13%)	76 (26%)	0.41 (0.14–1.23)	0.11
SEIFA index decile	3 (2–6)	2 (1–5)	1.09 (0.93–1.26)	0.29
Wet season presentation	26 (84%)	209 (72%)	2.01 (0.75–5.43)	0.17
Acute presentation	30 (97%)	254 (88%)	4.25 (0.56–32.13)	0.16
Diabetes mellitus	12 (39%)	151 (52%)	0.58 (0.27–1.24)	0.16
Macrovascular disease	9 (29%)	61 (21%)	1.54 (0.67–3.51)	0.31
Chronic kidney disease	3 (10%)	40 (14%)	0.67 (0.19–2.31)	0.53
Chronic lung disease	8 (26%)	75 (26%)	1.00 (0.43–2.32)	1.00
Hazardous alcohol use	8 (26%)	95 (33%)	0.71 (0.31–1.66)	0.43
Malignancy	**7 (23%)**	**30 (10%)**	**2.53 (1.00–6.36)**	0.049^**b**^
Immunosuppression	5 (16%)	37 (13%)	1.31 (0.48–3.64)	0.60
No risk factors^c^	5 (16%)	31 (11%)	1.61 (0.58–4.49)	0.37
Statin therapy^d^	5 (16%)	95 (33%)	0.39 (0.15–1.06)	0.07^b^
Bacteraemic	26 (84%)	199 (69%)	2.38 (0.88–6.39)	0.09^b^
Pulmonary involvement	**30 (97%)**	**224 (77%)**	**8.83 (1.18–66.05)**	0.03^**b**^
Bone or joint involvement	0	31 (11%)	—	—
Muscle involvement	0	18 (6%)	—	—
Skin and soft tissue involvement	4 (13%)	43 (15%)	0.85 (0.28–2.55)	0.77
Genitourinary tract involvement	5 (16%)	58 (20%)	0.77 (0.28–2.09)	0.61
Liver involvement	1 (3%)	19 (7%)	0.48 (0.06–3.68)	0.48
Spleen involvement	0	16 (6%)	—	—
Prostatic involvement	1 (3%)	36 (12%)	0.24 (0.03–1.78)	0.16
Disseminated	1 (3%)	29 (10%)	0.30 (0.04–2.28)	0.25
Septic shock	**18 (58%)**	**33 (11%)**	**10.78 (4.84–24.00)**	<0.001^**b**^
ICU admission	**12 (39%)**	**50 (17%)**	**3.03 (1.38–6.64)**	**0.006**

*Note:* Statistically significant associations in univariable analysis are presented in bold.

Abbreviations: ICU, intensive care unit; SEIFA, Socioeconomic Indexes for Area.

^a^Univariable analysis.

^b^Included in multivariable analysis.

^c^No history of diabetes, hazardous alcohol use, chronic lung disease, chronic kidney disease, active malignancy or immunosuppression.

^d^Prescribed at presentation.

**Table 2:** Characteristics of the patients prescribed—and not prescribed—statin therapy.

Variable	Prescribed a statin^a^*n* = 100	Not prescribed a statin^a^*n* = 221	OR (95% CI)	*p* ^b^
Age	**65 (55–75)**	**52 (40–66)**	**1.05 (1.03–1.06)**	**< 0.0001**
Male sex	71 (71%)	141 (64%)	1.40 (0.83–2.32)	0.21
First Nations Australians	39 (39%)	91 (41%)	0.91 (0.56–1.48)	0.71
Remote residence	24 (24%)	56 (25%)	0.93 (0.54–1.61)	0.80
SEIFA index decile	2 (2–6)	2 (1–5)	1.02 (0.93–1.13)	0.66
Wet season presentation	70 (70%)	165 (75%)	0.79 (0.47–1.34)	0.38
Acute presentation	89 (89%)	195 (88%)	1.08 (0.51–2.28)	0.84
Diabetes mellitus	**68 (68%)**	**95 (43%)**	**2.81 (1.71–4.63)**	**< 0.0001**
Macrovascular disease	**43 (43%)**	**26 (12%)**	**5.60 (3.17–9.90)**	**< 0.0001**
Chronic kidney disease	**26 (26%)**	**17 (8%)**	**4.21 (2.16–8.21)**	**< 0.0001**
Chronic lung disease	29 (29%)	74 (33%)	1.46 (0.86–2.47)	0.16
Hazardous alcohol use	31 (31%)	52 (24%)	0.81 (0.49–1.36)	0.43
Malignancy	7 (7%)	30 (14%)	0.48 (0.20–1.13)	0.09
Immunosuppression	9 (9%)	33 (15%)	0.56 (0.26–1.23)	0.15
No risk factors^c^	6 (6%)	30 (14%)	0.41 (0.16–1.01)	0.05
Bacteraemic	76 (76%)	149 (67%)	1.53 (0.89–2.62)	0.12
Pulmonary involvement	79 (79%)	175 (79%)	0.99 (0.55–1.77)	0.97
Bone or joint involvement	11 (11%)	20 (9%)	1.24 (0.57–2.70)	0.58
Muscle involvement	6 (6%)	12 (5%)	1.11 (0.41–3.05)	0.84
Skin and soft tissue involvement	11 (11%)	36 (16%)	0.64 (0.31–1.31)	0.22
Genitourinary tract involvement	22 (22%)	41 (19%)	1.24 (0.69–2.22)	0.47
Liver involvement	2 (2%)	18 (8%)	0.23 (0.05–1.01)	0.052
Spleen involvement	4 (4%)	12 (5%)	0.72 (0.22–2.31)	0.59
Prostatic involvement	12 (12%)	25 (11%)	1.07 (0.51–2.22)	0.86
Multiple sites	25 (25%)	68 (31%)	0.75 (0.44–1.28)	0.29
Disseminated	8 (8%)	22 (10%)	0.79 (0.34–1.83)	0.58
Septic shock	13 (13%)	38 (17%)	0.72 (0.36–1.42)	0.34
ICU admission	18 (18%)	44 (20%)	0.88 (0.48–1.62)	0.69
Died before hospital discharge	5 (5%)	26 (12%)	0.39 (0.15–1.06)	0.07
Died at 12 months	16 (16%)	49 (22%)	0.66 (0.36–1.25)	0.20
Recurrence^d^	11 (11%)	11 (5%)	2.36 (0.99–5.64)	0.054

*Note:* Statistically significant associations in univariable analysis are presented in bold.

Abbreviations: ICU, intensive care unit; SEIFA, Socioeconomic Indexes for Area.

^a^At time melioidosis was diagnosed.

^b^Univariable analysis.

^c^No history of diabetes, hazardous alcohol use, chronic lung disease, chronic kidney disease, active malignancy or immunosuppression.

^d^Includes recrudescence, relapse and reinfection.

## Data Availability

Data cannot be shared publicly because of the Queensland Public Health Act 2005. Data are available from the Far North Queensland Human Research Ethics Committee (contact via email: FNQ_HREC@health.qld.gov.au) for researchers who meet the criteria for access to confidential data.
